# On the Algorithm Complexity of Generating Discrete Uniform Distribution from a Biased Coin

**DOI:** 10.3390/e28070817

**Published:** 2026-07-17

**Authors:** Mengqi Zhang, Guangqiang Teng, Xiaoyu Lei

**Affiliations:** 1 School of Economics, Peking University, Beijing 100871, China; 2 School of Mathematics, Harbin Institute of Technology, Harbin 150001, China; tenggq@alu.hit.edu.cn; 3 Department of Statistics, University of Wisconsin-Madison, 1205 University Ave, Madison, WI 53706, USA; xlei35@wisc.edu

**Keywords:** random number generation, biased coin, discrete uniform distribution, number theory, characteristic function, algorithm complexity, Monte Carlo methods, fairness in algorithms

## Abstract

Lei proposed an algorithm Algorithm $A_{3}$ in 2023 to generate an exact discrete uniform distribution from an unknown biased Bernoulli source. The present paper does not claim a new extraction algorithm. Its contributions are analytical: first, we provide a Fourier-analytic proof of the uniformity mechanism based on roots of unity and coefficient extraction; second, we derive explicit acceptance-probability and expected-runtime bounds, with a rigorous treatment of composite moduli; third, we show how independent accepted $A_{3}$ digits yield a continuous Uniform(0,1) limit through a base-*n* expansion and quantify the cost of finite-digit simulation.

## 1. Introduction

### 1.1. The Quest for Perfect Randomness in Imperfect Worlds

New theory and large experiments show that coin tossing is not perfectly fair in the strict physical sense. The dynamical model from [1] predicts a small precession in flight that makes the coin slightly more likely to land on the same face it started with. A large experiment led by the University of Amsterdam collected 350,757 flips and found the probability of landing on the starting face is about 50.8% with a 95% confidence interval of [0.506, 0.509], and this result closely matches the approximately 51% theoretical prediction while the overall heads versus tails frequencies remain symmetric with heads at roughly 50.0% and a 95% confidence interval of [0.498, 0.502]. No systematic bias exists toward heads or tails but a subtle starting-orientation effect does exist, meaning raw coin tossing is not ideal for those demanding exact fairness.

This mild but detectable bias shows the value of the method from [2] published in Electronic Communications in Probability in 2023. Even when the base bit source, which is the coin, has an unknown bias, one can generate exactly discrete-uniform random numbers without parameters and with proof. The algorithm uses n tosses to form the head count *N* and a rank-sum *R* and it outputs *R* mod *n* whenever gcd(N,n)=1; it adds an independent random cyclic shift if the flips are independent but not identically distributed, and this process yields an exactly uniform value on {0,…,n−1}. Even with a starting-orientation bias in physical tossing, this number-theoretic–probabilistic “uniformization” delivers strictly fair *n*-ary outcomes that are useful for instructional labs, low-cost randomness acquisition and any application that needs verifiable fairness.

### 1.2. Historical Context and Theoretical Foundations

The present problem is related to broader literature on extracting unbiased randomness from biased sources, beginning with von Neumann’s extractor [3] and including refinements by Elias [4], Knuth-Yao [5], and Peres [6]. These methods optimize different efficiency or source-assumption criteria.

The mathematical problem of making fair random bits from biased coins has a long history. It started with von Neumann’s important 1951 work [3]. For a coin with unknown bias *p*, von Neumann suggested a simple algorithm. Flip the coin twice. Use the first flip as the result if the outcomes are different. Discard the two flips and try again if they are the same. This simple process gets exactly one fair bit from two biased flips. It has a variable cost and the expected runtime is 1/(2p(1−p)) flips per output bit.

Dijkstra [7] extended this framework to prime numbers. He created an algorithm that generates a uniform distribution on *p* elements from *p* coin flips. This method uses the algebraic structure of cyclic groups and lexicographic ranking within orbits made by cyclic shifts. The primality of *p* ensures each orbit has exactly *p* distinct configurations and this allows uniform sampling.

Lei [2] simplified this method by using rank sums modulo *p* instead of complex orbit analysis, replacing the latter with a straightforward modular computation. His simplification laid the groundwork for generalizing the method to any integer *n*, using a number-theoretic approach that combines ideas from probability theory and algebra. Lei’s algorithm $A_{3}$ is distinctive in that it produces exact *n*-ary uniform variables directly from a Bernoulli source through a number-theoretic acceptance condition. Our contribution is to analyze this mechanism using Fourier coefficients and to quantify the acceptance cost.

### 1.3. Applications in Modern Data Science

The algorithm’s importance extends beyond theoretical interest to practical applications across modern data science:

Statistical computing: The generation of non-uniform deviates via inversion, acceptance-rejection, or MCMC fundamentally relies on a source of i.i.d. uniform draws. By supplying such draws from an arbitrarily biased Bernoulli stream, $A_{3}$ immunises these procedures against source-level imperfections. In particular, Metropolis-Hastings samplers require proposal steps with exact uniformity to preserve detailed balance; the proposed method provides a parameter-free guarantee that is otherwise difficult to achieve without knowledge of the physical bias.

Machine Learning and Randomized Algorithms:Modern machine learning uses more and more randomness. It uses randomness for initialization (neural network weights), regularization (dropout), and optimization (stochastic gradient descent). This algorithm has provable fairness. It makes sure that these random parts do not add unintended biases. Ensemble methods like random forests use bootstrap sampling and random feature selection. This algorithm ensures that each data point and feature is selected with equal probability.

Reinforcement Learning: In Reinforcement learning (RL) and bandit algorithms, exploration strategies such as ϵ-greedy or Thompson sampling algorithms need uniform sampling to explore state spaces and *K*-arms; see [8]. A biased generator can cause systematic under-exploration of some action trajectories. MCMC inference uses an acceptance ratio. This ratio needs to compare a computed likelihood with a uniform variable u∼U[0,1]. The bias in *u* breaks the detailed balance condition. This means the chain converges to the wrong stationary distribution. We extend $A_{3}$ to the continuous domain (Section 5) to fix this verification gap.

Cryptography and Security: Cryptographic protocols need high-quality randomness for key generation, nonce creation and probabilistic encryption. Cryptographic applications usually need stronger security guarantees than physical coin tossing alone can provide. This algorithm gives a valuable post-processing step. It helps distill entropy from imperfect sources in randomness extractors [9].

A concrete use case is finite experimental randomization from a simple biased physical or hardware Bernoulli source. Suppose a laboratory device produces binary events with unknown but stable bias *p*, and the experiment requires exact assignment among *n* treatment arms. Estimating *p* and then correcting the source introduces calibration error, whereas $A_{3}$ gives an exact uniform *n*-ary output under the i.i.d. Bernoulli model. This is the regime in which the algorithm is preferable: exact finite-sample *n*-ary uniformization is required, the source bias is unknown, and cryptographic security is not the primary objective.

### 1.4. Contributions and Paper Overview

In summary, the contribution of this paper is as follows: (1) We provide a characteristic-function proof of Lei’s algorithm $A_{3}$ [2] using roots of unity and coefficient extraction. (2) We prove explicit acceptance-probability and expected-runtime bounds, including the composite-modulus case. (3) We use independent accepted $A_{3}$ digits to construct a continuous Uniform(0,1) limit and quantify finite-digit runtime and approximation error.

The specific contributions are as follows:

1. A Novel Characteristic Function Proof (Section 3): We use a rigorous analytic proof with Characteristic Functions, which are Fourier analysis on $Z_{n}$, instead of the combinatorial group-action proof from the original paper. This proof is more robust and directly links the number-theoretic condition gcd(N,n)=1 to the vanishing of Fourier coefficients at non-zero frequencies.
2. Complexity Analysis (Section 4): We establish rigorous bounds on the algorithm’s expected runtime. We show the expected number of coin flips is O(n) when *n* is prime and O(nloglogn) when *n* is composite. We find explicit constants with Euler’s totient function ϕ(n) and give detailed asymptotic expansions using analytic number theory tools.
3. Extension to Continuous Uniform Distribution (Section 5): We show $A_{3}$ can be used to generate continuous uniform random variables on [0,1] with base-*n* expansion. We prove convergence with characteristic function arguments and give error bounds for practical use.

The paper is organized as follows: Section 2 formally presents $A_{3}$ and establishes notation. Section 3 provides the characteristic function proof of uniformity. Section 4 analyzes computational complexity with detailed asymptotic expansions. Section 5 extends this method to the generation of continuous uniform distributions. Section 6 concludes with discussion and future directions.

## 2. Preliminaries and Algorithm Formulation

We operate within a standard probability space (Ω,F,P). Let N={1,2,…} and $N_{0}=\left\{0,1,\ldots \right\}$.

### 2.1. Model and Sufficient Statistics

**Assumption** **1** (Bernoulli Source)**.** *Let $X_{0},X_{1},\ldots$ be a sequence of independent and identically distributed (i.i.d.) random variables taking values in {0,1}. We denote the success probability by $p=P(X_{i}=1)$ and the failure probability by $q=P(X_{i}=0)=1-p$. We assume p∈(0,1) is unknown.*

We denote a sequence of *n* flips as a random vector $X^{n}=\left(X_{0},X_{1},\ldots ,X_{n-1}\right)\in {\left\{0,1\right\}}^{n}$.

The algorithm relies on two statistics of the sample $X^{n}$: the Hamming weight (head count) and a weighted positional sum (rank sum).

**Definition 1** (Head Count)**.** *For a vector $X^{n}$, the head count is defined as:*

(1) $$N\left(X^{n}\right)=\sum_{i=0}^{n-1}X_{i}.$$

**Definition 2** (Rank Sum)**.** *The rank sum is defined as the sum of indices where heads occur:*

(2) $$R\left(X^{n}\right)=\sum_{i=0}^{n-1}i\cdot X_{i}.$$

**Definition 3** (Coprimality)**.** *Let gcd(x,y) denote the greatest common divisor of integers x and y. We say N and n are coprime if gcd(N,n)=1. The set of integers in {0,…,n−1} coprime to n forms the multiplicative group of integers modulo n, denoted by $Z_{n}^{\times }$. The size of this group is given by Euler’s totient function ϕ(n).*

### 2.2. Algorithm $A_{3}$

The core algorithm proposed in [2] is a rejection sampling procedure. It generates batches of size *n* and accepts a batch only if the head count *N* is coprime to the batch size *n* (see Algorithm 1).
**Algorithm 1.** Discrete Uniform Generation**Require:** Sequence of biased coin flips $X_{i}∼\mathrm{Ber}\left(p\right)$. Target size n∈N,n≥2.**Ensure:** Random variable *Y* uniformly distributed on {0,1,…,n−1}.  1:Initialize valid ← **false**.  2:**while** not valid **do**  3:      Sample *n* independent flips $X^{n}=\left(X_{0},\ldots ,X_{n-1}\right)$.  4:      Calculate $N=\sum_{i=0}^{n-1}X_{i}$.  5:      **if** gcd(N,n)==1 **then**  6:         Calculate $R=\sum_{i=0}^{n-1}i\cdot X_{i}$.  7:         Set Y=R(modn).  8:         valid ← **true**.  9:      **else** 10:        Discard $X^{n}$ and restart. 11:     **end if** 12:**end while** 13:**return** *Y*.

### 2.3. Illustrative Examples

**Example 1** (n=2: Connection to von Neumann’s Algorithm)**.** *For n=2, $A_{3}$ reduces to a variant of von Neumann’s original procedure. Consider all possible outcomes:*

- *(0,0): N=0, gcd(0,2)=2>1, reject*
- *(1,1): N=2, gcd(2,2)=2>1, reject*
- *(0,1): N=1, gcd(1,2)=1, R=0·0+1·1=1, output 1mod2=1*
- *(1,0): N=1, gcd(1,2)=1, R=0·1+1·0=0, output 0mod2=0*

*The algorithm accepts exactly when the two flips differ, producing outputs 0 for (H,T) and 1 for (T,H), matching von Neumann’s procedure.*

**Example 2** (n=6: Composite Case)**.** *Consider n=6 and the outcome $X^{6}=\left(1,0,1,0,1,0\right)$:*

- *Head count: N=1+0+1+0+1+0=3*
- *gcd(3,6)=3>1, so reject*

*Now consider $X^{6}=\left(1,1,0,0,0,1\right)$:*

- *Head count: N=1+1+0+0+0+1=3*
- *gcd(3,6)=3>1, reject again*

*Finally, consider $X^{6}=\left(1,0,0,1,0,0\right)$:*

- *Head count: N=1+0+0+1+0+0=2*
- *gcd(2,6)=2>1, reject*

*These examples illustrate that many outcomes are rejected when n has multiple prime factors.*

## 3. Characteristic Function Proof of Uniformity

The algorithm admits a natural algebraic interpretation. Consider the random variable $Y_{i}\in \left\{\bar{0},\bar{1}\right\}$ where $\bar{0}$ represents tails and $\bar{1}$ represents heads. Then, the *n* flips correspond to a random vector $\left(Y_{0},\ldots ,Y_{n-1}\right)$ in $Z_{n}^{n}$. The head count condition gcd(N,n)=1 ensures that the sum $\sum_{i=0}^{n-1}Y_{i}$ generates the entire group $Z_{n}$, while the rank sum condition Rmodn provides a linear combination that, under this coprimality condition, yields uniform distribution.

**Theorem** **1.** *Let $X_{0},\ldots ,X_{n-1}$ be identically distributed Bernoulli variables with the probability of success p∈(0,1) and the probability of failure q=1−p. Let $N=\sum_{i=0}^{n-1}X_{i}$ and $R=\sum_{i=0}^{n-1}iX_{i}$. For any integer c such that gcd(c,n)=1, the conditional distribution of R(modn) given N=c is uniform on {0,…,n−1}.*

### 3.1. Refined Proof: Group Action Method

**Proof.**  Let $\Omega ={\left\{0,1\right\}}^{n}$ be the sample space of outcomes $x=(x_{0},\ldots ,x_{n-1})$. Consider the cyclic shift operator T:Ω→Ω defined by

$$T\left(x_{0},\ldots ,x_{n-1}\right)=\left(x_{1},\ldots ,x_{n-1},x_{0}\right).$$

Since the random variables $X_{0},\ldots ,X_{n-1}$ are i.i.d., the probability measure *P* on Ω is invariant under *T*; that is, P(X=x)=P(X=Tx) for all x∈Ω.

We examine the behavior of the statistics *N* and *R* under this transformation. The total count is invariant:

$$N\left(Tx\right)=\sum_{i=0}^{n-1}x_{i}=N\left(x\right).$$

For the rank sum *R*, observing that indices shift by −1(modn), we compute:

$$\begin{array}{cc} R(Tx) & =\sum_{i=0}^{n-2}ix_{i+1}+\left(n-1\right)x_{0}\\ & =\sum_{j=1}^{n-1}\left(j-1\right)x_{j}+\left(n-1\right)x_{0}=\sum_{j=1}^{n-1}jx_{j}-\sum_{j=1}^{n-1}x_{j}+\left(n-1\right)x_{0}\\ & =\sum_{j=0}^{n-1}jx_{j}-\left(N\left(x\right)-x_{0}\right)+nx_{0}-x_{0}\\ & =R\left(x\right)-N\left(x\right)+n\;x_{0}\\ & \equiv R(x)-N(x)\;(\mathrm{mod}\;n). \end{array}$$

By induction, for any $k\in Z_{\geq 0}$, the *k*-th iteration of the shift satisfies

$$R\left(T^{k}x\right)\equiv R\left(x\right)-kN\left(x\right)\;\left(\mathrm{mod}\;n\right).$$

Consider the set of outcomes $S_{c}=\left\{x\in \Omega :N\left(x\right)=c\right\}$ for a fixed count *c* satisfying gcd(c,n)=1. The operator *T* partitions $S_{c}$ into disjoint orbits. For any $x\in S_{c}$, the orbit is $O_{x}=\left\{x,Tx,\ldots ,T^{n-1}x\right\}$.

Because gcd(c,n)=1, the map k↦−kc(modn) is a bijection on $Z_{n}$. Consequently, the set of values $\left\{R\left(y\right)\;\left(\mathrm{mod}\;n\right):y\in O_{x}\right\}$ is exactly ${\left\{R\left(x\right)-kc\;\left(\mathrm{mod}\;n\right)\right\}}_{k=0}^{n-1}$, which is a permutation of {0,…,n−1}.

Since the measure is invariant under *T*, every element in a given orbit $O_{x}$ has equal probability. Thus, conditional on falling into a specific orbit, R(modn) is uniform. Since $S_{c}$ is a disjoint union of such orbits, R(modn) is uniform on $S_{c}$. □

This algebraic perspective connects the algorithm to deeper number-theoretic structures and motivates the characteristic function approach developed. We now use roots of unity to characterize the discrete uniform distribution on the set {0,1,…,n−1}.

**Lemma** **1.** *Let Z be a random variable supported on {0,1,…,n−1} and write*

$$\phi_{Z}\left(t\right)\;=\;E\;\left[e^{itZ}\right],\;t\in R.$$

*Let $\omega :=e^{2\pi i/n}$ and define the discrete Fourier coefficients*

$$\hat{p}\left(m\right)\;:=\;E\;\left[\omega^{\;mZ}\right]\;=\;\phi_{Z}\;\left(\frac{2\pi m}{n}\right),\;m=0,1,\ldots ,n-1.$$

*Then the following are equivalent:*

*1*. *Z is uniformly distributed on {0,1,…,n−1}.*
*2*. *$\hat{p}\left(0\right)=1$ and $\hat{p}\left(m\right)=0$ for all m=1,…,n−1; equivalently*

$\phi_{Z}\;\left(\frac{2\pi m}{n}\right)=\left\{\begin{array}{cc} 1, & m=0,\\ 0, & m=1,\ldots ,n-1. \end{array}\right.$

**Proof.**  *(1 ⇒ 2).* If *Z* is uniform on {0,1,…,n−1}, then P(Z=k)=1/n for all *k* and

$$\hat{p}\left(m\right)=\frac{1}{n}\sum_{k=0}^{n-1}\omega^{\;mk}.$$

For m=0 this is 1. For m=1,…,n−1 we use the geometric series:

$$\sum_{k=0}^{n-1}\omega^{\;mk}=\frac{1-{\left(\omega^{m}\right)}^{n}}{1-\omega^{m}}=\frac{1-1}{1-\omega^{m}}=0,$$

so $\hat{p}\left(m\right)=0$. Because $\hat{p}\left(m\right)=\phi_{Z}\left(2\pi m/n\right)$, the stated values of $\phi_{Z}$ follow.

*(2 ⇒ 1).* Let $p_{k}=P\left(Z=k\right)$, k=0,…,n−1, and consider the discrete Fourier transform (DFT)

$$\hat{p}\left(m\right)=\sum_{k=0}^{n-1}p_{k}\;\omega^{\;mk},\;m=0,\ldots ,n-1.$$

On the finite abelian group $Z_{n}$, the DFT is invertible with the inversion formula

(3) $$p_{k}=\frac{1}{n}\sum_{m=0}^{n-1}\hat{p}\left(m\right)\;\omega^{-mk},\;k=0,\ldots ,n-1,$$

which follows from the orthogonality of *n*th roots of unity:

$$\frac{1}{n}\sum_{m=0}^{n-1}\omega^{\;m(k-\ell )}=\left\{\begin{array}{cc} 1, & k=\ell ,\\ 0, & k\neq \ell . \end{array}\right.$$

Now apply (3) with the assumption $\hat{p}\left(0\right)=1$ and $\hat{p}\left(m\right)=0$ for m≥1:

$$p_{k}=\frac{1}{n}\left(\hat{p}\left(0\right)\;\omega^{0}+\sum_{m=1}^{n-1}\hat{p}\left(m\right)\;\omega^{-mk}\right)=\frac{1}{n}\;.$$

Hence $p_{k}=1/n$ for all *k*, i.e., *Z* is uniform on {0,1,…,n−1}. □

While the group-action proof is combinatorially elegant, the characteristic-function approach reveals directly how gcd(N,n)=1 forces the Fourier coefficients to vanish. The argument proceeds as follows.

### 3.2. New Proof: Characteristic Function Method

**Proof.**  Let $\omega =e^{2\pi i/n}$. To establish uniformity on $Z_{n}$, it suffices to show that the conditional characteristic function of *R* vanishes at all non-zero frequencies. Specifically, we must show that for all k∈{1,…,n−1}:

$$E[\omega^{kR}|N=c]=0.$$

Since gcd(c,n)=1 implies 1≤c≤n−1 and p∈(0,1), we have $P\left(N=c\right)=\left(\begin{array}{c} n\\ c \end{array}\right)p^{c}{\left(1-p\right)}^{c}>0$. Hence the conditional expectation is well-defined by

$$\frac{E[\omega^{kR}1_{\left\{N=c\right\}}]}{P(N=c)}.$$

We analyze the numerator. Consider the joint characteristic function (CF) of (R,N), defined for z∈C as:

$$\Psi_{k}\left(z\right)=E\left[\omega^{kR}z^{N}\right]=E\left[\prod_{j=0}^{n-1}\omega^{kjX_{j}}z^{X_{j}}\right].$$

Since $\left|\omega^{kR}z^{N}\right|\leq {\left|z\right|}^{N}$ and $E\left[{\left|z\right|}^{N}\right]={\left(1-p+p\left|z\right|\right)}^{n}<\infty$ for |z|≤1, the series converges absolutely and coefficient extraction is valid.

By independence, the expectation factors:

$$\Psi_{k}\left(z\right)=\prod_{j=0}^{n-1}E\left[{\left(\omega^{kj}z\right)}^{X_{j}}\right]=\prod_{j=0}^{n-1}\left(q+pz\omega^{kj}\right).$$

where $p=P\left(X_{j}=1\right)$ and q=1−p.

Let $d_{k}=\gcd\left(k,n\right)$ and $m_{k}=n/d_{k}$. The sequence of exponents ${\left\{kj\;\left(\mathrm{mod}\;n\right)\right\}}_{j=0}^{n-1}$ traverses the values $\left\{0,d_{k},2d_{k},\ldots ,\left(m_{k}-1\right)d_{k}\right\}$, each repeated $d_{k}$ times. With $\eta =\omega^{d_{k}}$, a primitive $m_{k}$-th root of unity,

$$\Psi_{k}\left(z\right)={\left\{\prod_{r=0}^{m_{k}-1}\left(q+pz\eta^{r}\right)\right\}}^{d_{k}}={\left\{q^{m_{k}}-{\left(-pz\right)}^{m_{k}}\right\}}^{d_{k}}.$$

Therefore $\Psi_{k}\left(z\right)$ is a polynomial in $z^{m_{k}}$. Consequently $\left[z^{c}\right]\Psi_{k}\left(z\right)=0$ unless $m_{k}|c$. If 1≤k≤n−1, then $m_{k}>1$ and $m_{k}|n$; since gcd(c,n)=1, $m_{k}∤c$. Hence $\left[z^{c}\right]\Psi_{k}\left(z\right)=0$, and the nonzero Fourier coefficients vanish. We have:

$$E\left[\omega^{kR}1_{\left\{N=c\right\}}\right]=\left[z^{c}\right]\Psi_{k}\left(z\right).$$

where the notation $\left[z^{c}\right]\Psi_{k}\left(z\right)$ is the coefficient of the term $z^{c}$ in the series expansion of $\Psi_{k}\left(z\right)$. Since

$$\begin{array}{c} E\left[\omega^{kR}1_{\left\{N=c\right\}}\right]=\sum_{r}\sum_{m}P\left(R=r,N=m\right)\cdot \omega^{kr}\cdot 1_{\left\{m=c\right\}}=\sum_{r}P\left(R=r,N=c\right)\cdot \omega^{kr}\cdot 1 \end{array}$$

Given the hypothesis gcd(c,n)=1, *c* cannot be a multiple of $m_{k}=n/\gcd\left(k,n\right)$ unless $m_{k}=1$. If k∈{1,…,n−1}, then gcd(k,n)<n, implying $m_{k}>1$. Since $m_{k}$ is a divisor of *n* greater than 1, and gcd(c,n)=1, it follows that $m_{k}∤c$. Therefore, $\left[z^{c}\right]\Psi_{k}\left(z\right)=0$ for all k≢0(modn).

The conditional characteristic function is thus:

$$E\left[\omega^{kR}|N=c\right]=\left\{\begin{array}{cc} 1 & \mathrm{if}\;k\equiv 0\;(\mathrm{mod}\;n),\\ 0 & \mathrm{if}\;k≢0\;(\mathrm{mod}\;n). \end{array}\right.$$

By the uniqueness of the CF, *R* is uniformly distributed on {0,…,n−1} conditional on N=c. □

## 4. Complexity Analysis of Algorithm $A_{3}$

We analyze the sample complexity of Algorithm $A_{3}$, defined as the expected number of coin flips required to generate a single output sample.

**Theorem 2** (Expected Complexity)**.** *Let p∈(0,1) be the success probability of the biased coin. Let $T_{n}$ be the random variable representing the total number of coin flips performed by Algorithm $A_{3}$ to generate one output in {0,…,n−1}.*

*The expected number of flips satisfies the following asymptotic bound as n→∞:*

$$E\left[T_{n}\right]=O\left(n\log\log n\right).$$

*Furthermore, if n is prime, the complexity is strictly linear:*

$$E\left[T_{n}\right]=n+o\left(1\right).$$

**Proof.**  Algorithm $A_{3}$ operates in independent trials. In each trial, a batch of *n* coin flips is generated. Let $X^{\left(n\right)}=\left(X_{0},\ldots ,X_{n-1}\right)$ be the sequence of outcomes in a single batch, and let $N=\sum_{i=0}^{n-1}X_{i}$ be the number of heads. The batch is accepted if and only if gcd(N,n)=1.

Let $\alpha_{n}$ denote the probability of acceptance in a single trial:

αn=P(gcd(N,n)=1)=∑0≤k≤ngcd(k,n)=1nkpk(1−p)n−k.

Since trials are independent, the number of trials *M* required to obtain a valid output follows a Geometric distribution with parameter $\alpha_{n}$. The total number of flips is $T_{n}=n\cdot M$. By the properties of the Geometric distribution ($E\left[M\right]=1/\alpha_{n}$), the expected number of flips is:

$$E\left[T_{n}\right]=\frac{n}{\alpha_{n}}.$$

Case 1: *n* is Prime

If *n* is prime, gcd(k,n)=1 for all k∈{1,…,n−1}. The condition fails only if N=0 or N=n.

$$\alpha_{n}=1-P\left(N=0\right)-P\left(N=n\right)=1-{\left(1-p\right)}^{n}-p^{n}.$$

For any fixed p∈(0,1), the terms ${\left(1-p\right)}^{n}$ and $p^{n}$ decay exponentially to 0. Thus, $\lim_{n\to \infty }\alpha_{n}=1$.

$$E\left[T_{n}\right]\approx \frac{n}{1}=O\left(n\right).$$

Case 2: *n* is Composite (General Case)

We estimate $\alpha_{n}$ for composite *n*. As n→∞, the distribution of the sum *N* modulo *d* approaches a uniform distribution on {0,…,d−1} when *d* is any fixed divisor of *n*.

The probability that *N* is coprime to *n* behaves asymptotically like the density of integers coprime to *n* in the range [0,n−1] and this density is given by Euler’s totient function ratio $\frac{\phi (n)}{n}$:

$$\alpha_{n}=\frac{\phi (n)}{n}+r_{n},\;\left|r_{n}\right|\leq C_{p}\frac{\tau (n)}{\sqrt{n}}.$$

Thus, for fixed p∈(0,1) and sufficiently large *n*, $\alpha_{n}\geq \frac{1}{2}\phi \left(n\right)/n$. This is the only estimate needed for the complexity upper bound.

The relevant lemmas and proofs can be found in Appendix A. Substituting this into the expectation:

$$E\left[T_{n}\right]=\frac{n}{\alpha_{n}}\leq \frac{2n}{\phi (n)/n}=\frac{2n^{2}}{\phi (n)}.$$

By Landau’s theorem (see Theorem 328 of [10]), n/ϕ(n)=O(loglogn), hence

$$E\left[T_{n}\right]=O\left(n\log\log n\right).$$

The prime case remains sharper. If *n* is prime, the only rejected head counts are 0 and *n*, so

$$\alpha_{n}=1-q^{n}-p^{n},\;E\left[T_{n}\right]=\frac{n}{1-q^{n}-p^{n}}=n+O\left(n\left(p^{n}+q^{n}\right)\right)=n+o\left(1\right).$$

   □

According to Theorem 2, when *n* is prime, the expected complexity of Algorithm $A_{3}$ achieves a strict linear bound $E\left[T_{n}\right]=n+o\left(1\right)$. This implies that the algorithm’s acceptance probability $\alpha_{n}$ is approximately 1/n (as derived from $T_{n}=n/\alpha_{n}$), thereby achieving an optimal linear time cost for generating a single output.

For the composite case, our analysis using Fourier methods and number theory demonstrates that the acceptance probability satisfies $\alpha_{n}=\frac{\phi (n)}{n}+O_{p}\left(\frac{\tau (n)}{\sqrt{n}}\right)$, where ϕ(n) is Euler’s totient function and τ(n) is the divisor function. By leveraging the properties $\tau \left(n\right)=n^{o(1)}$ and n/ϕ(n)=O(loglogn), we establish the lower bound $\alpha_{n}\geq \frac{1}{2}\cdot \frac{\phi (n)}{n}$. This directly leads to the expected complexity bound of O(nloglogn).

This refined analysis not only explains the root cause of the complexity difference between prime and composite cases but also rigorously demonstrates, through a proven lower bound on the acceptance probability, that the O(nloglogn) complexity for composite *n* is indeed tight.

**Remark** **1.** *The appearance of ϕ(n)/n has a simple interpretation. A batch is accepted precisely when its head count lies in a residue class coprime to n. Among the n residues modulo n, exactly ϕ(n) are coprime to n. Thus ϕ(n)/n is the ideal acceptance density. The Moebius–Fourier calculation quantifies the deviation of the binomial head-count distribution from this ideal residue density.*

## 5. Continuous Complexity Analysis of Algorithm $A_{3}$

Having established the strict uniformity of the algorithm through a purely analytic characteristic function proof and derived tight asymptotic bounds on its expected runtime, we proceed to extend the method to the continuous uniform distribution in this section. We utilize the discrete extraction method from Theorem 3 to construct a random variable *U* on the continuous interval [0,1].

**Definition** **4** (Base-*n* Digit Generator)**.** *Let S be a stream of i.i.d. Bernoulli trials. We parse S into disjoint blocks of length n. For each block j, we compute the total sum $N_{j}$ and rank sum $R_{j}$.*

- *If $\gcd(N_{j},n)=1$, we accept the block and output the digit $D_{k}=R_{j}\;\left(\mathrm{mod}\;n\right)$.*
- *If $\gcd(N_{j},n)\neq 1$, we discard the block.*

*Let $D_{1},D_{2},\ldots$ be the sequence of successfully generated digits. By Theorem 3 and the independence of the blocks, ${\left\{D_{k}\right\}}_{k\geq 1}$ is a sequence of i.i.d. variables uniformly distributed on {0,1,…,n−1}.*

Base-*n* expansions are not unique on the countable set of *n*-adic rationals, where a terminating expansion can also be written with an eventually all-(n−1) tail. We adopt the canonical convention that excludes the eventually all-(n−1) representation. The digit sequence generated by independent accepted A3 blocks is almost surely not eventually constant equal to n−1, so the ambiguity has probability zero and does not affect the distributional conclusion.

**Theorem** **3** (Continuous Uniformity)**.** *Let U be the random variable defined by the base-n expansion using the digits generated above:*

$$U=\sum_{k=1}^{\infty }\frac{D_{k}}{n^{k}}.$$

*Then U follows a continuous uniform distribution on* [0,1]*, i.e.,* U∼Uniform[0,1]*.*

**Proof.**  We use the method of Characteristic Functions. The characteristic function of the target distribution Uniform[0,1] is:

$$\phi_{\mathrm{target}}\left(t\right)=\int_{0}^{1}e^{itx}\;dx=\frac{e^{it}-1}{it}.$$

We must show that $\phi_{U}\left(t\right)=E\left[e^{itU}\right]$ is equal to $\phi_{\mathrm{target}}\left(t\right)$.

Case 1: Characteristic Function of a Single Digit

Since $D_{k}$ is uniform on {0,…,n−1}, its characteristic function is:

$$E\left[e^{i\xi D_{k}}\right]=\frac{1}{n}\sum_{j=0}^{n-1}e^{i\xi j}=\frac{1}{n}\frac{e^{in\xi }-1}{e^{i\xi }-1}.$$

Case 2: Characteristic Function of the Partial Sum

Let $U_{M}=\sum_{k=1}^{M}D_{k}n^{-k}$ be the partial sum of the first *M* digits. Since the digits are independent, the characteristic function of the sum is the product of the characteristic functions of the individual terms. The *k*-th term is $X_{k}=D_{k}n^{-k}$.

$$\phi_{U_{M}}\left(t\right)=E\left[\exp\left(it\sum_{k=1}^{M}\frac{D_{k}}{n^{k}}\right)\right]=\prod_{k=1}^{M}E\left[e^{i\left(t/n^{k}\right)D_{k}}\right].$$

Substituting $\xi =t/n^{k}$ into the single digit formula:

$$E\left[e^{i\left(t/n^{k}\right)D_{k}}\right]=\frac{1}{n}\frac{e^{in(t/n^{k})}-1}{e^{i(t/n^{k})}-1}=\frac{1}{n}\frac{e^{it/n^{k-1}}-1}{e^{it/n^{k}}-1}.$$

The preceding calculation assumes t≠0 because the displayed telescoping quotient contains $e^{it/n^{M}}-1$ in the denominator. The point t=0 is immediate: for every *M*, $\phi_{U_{M}}\left(0\right)=1$, and the characteristic function of Uniform[0,1] also equals 1 at zero. We therefore define $\left(e^{it}-1\right)/\left(it\right)$ at t=0 by continuity. Hence the characteristic functions converge for all t∈R.

The product over k=1 to *M* telescopes:

$$\begin{array}{cc} \phi_{U_{M}}\left(t\right) & =\left(\frac{1}{n}\frac{e^{it}-1}{e^{it/n}-1}\right)\cdot \left(\frac{1}{n}\frac{e^{it/n}-1}{e^{it/n^{2}}-1}\right)\cdots \left(\frac{1}{n}\frac{e^{it/n^{M-1}}-1}{e^{it/n^{M}}-1}\right)\\ & =\frac{1}{n^{M}}\frac{e^{it}-1}{e^{it/n^{M}}-1}. \end{array}$$

Case 3: Convergence to the Continuum

We take the limit as M→∞. Consider the denominator term $e^{it/n^{M}}-1$. Using the Taylor expansion $e^{x}\approx 1+x$ for small *x*:

$$e^{it/n^{M}}-1∼\frac{it}{n^{M}}\;\mathrm{as}\;M\to \infty .$$

Substituting this back into the expression for $\phi_{U_{M}}\left(t\right)$:

$$\lim_{M\to \infty }\phi_{U_{M}}\left(t\right)=\lim_{M\to \infty }\frac{1}{n^{M}}\frac{e^{it}-1}{\left(it/n^{M}\right)}=\frac{e^{it}-1}{it}.$$

The limit matches $\phi_{\mathrm{target}}\left(t\right)$. By the Lévy Continuity Theorem, $U_{M}$ converges in distribution to *U*, and *U* is uniformly distributed on [0,1]. □

For finite-precision simulation, the generator outputs $U_{M}=\sum_{k=1}^{M}D_{k}n^{-k}$. Since each digit requires one accepted A3 block and each block has acceptance probability $\alpha_{n}$, the expected number of biased flips is $Mn/\alpha_{n}$. Hence the finite-precision runtime is Mn(1+o(1)) for prime *n* and O(Mnloglogn) for general *n*. Under the canonical coupling with the infinite expansion, $|U-U_{M}|\;\leq \;n^{-M}$.

In fact, let ${\left(D_{k}\right)}_{k\geq 1}$ be the i.i.d. digits generated by accepted A3 blocks. Define

$$U=\sum_{k=1}^{\infty }D_{k}n^{-k},\;U_{M}=\sum_{k=1}^{M}D_{k}n^{-k}.$$

Then U∼Uniform[0,1] and

$$0\leq U-U_{M}\leq \sum_{k=M+1}^{\infty }\left(n-1\right)n^{-k}=n^{-M}.$$

The expected number of biased coin flips required to generate $U_{M}$ is

$$E\left[T_{n,M}\right]=M\frac{n}{\alpha_{n}},$$

which is Mn(1+o(1)) for prime *n* and O(Mnloglogn) for general *n*.

The algorithm does not require storing the entire block. During the scan of a block, *N* and Rmodn can be updated incrementally. Computing gcd(N,n) by the Euclidean algorithm adds only logarithmic arithmetic overhead. Therefore the dominant cost remains the generation of Bernoulli trials, while the modular arithmetic and gcd check are standard low-order implementation costs.

## 6. Conclusions

This paper has provided a comprehensive theoretical treatment of Lei’s algorithm $A_{3}$ for extracting exact discrete uniform distributions from biased Bernoulli trials, and has extended its reach to continuous uniform generation. Three primary contributions were presented. First, in terms of algorithm validity verification, we abandon the traditional group-theoretic proof framework and propose a novel analytic proof based on characteristic functions and roots of unity. Second, regarding sample complexity analysis, we derive the first explicit asymptotic bounds for Algorithm $A_{3}$ by combining properties of the binomial distribution, Euler’s totient function ϕ(n), and classical results from analytic number theory. Third, in the extension to continuous uniform distributions, we construct a Base-*n* Digit Generator using the discrete uniform digits output by $A_{3}$.

From a methodological standpoint, the characteristic-function perspective not only simplifies the proof but also suggests possible generalizations to other group structures. The complexity analysis, albeit asymptotic, confirms that the rejection penalty for composite moduli is mild—only a logarithmic factor—and is unlikely to hinder practical deployment.

Future research directions can focus on three aspects: first, optimizing the rejection sampling strategy to reduce the discard rate of composite *n*, especially for large *n* with multiple prime factors; second, extending the algorithm to non-i.i.d. Bernoulli sources (e.g., sources with weak dependence) while maintaining uniformity guarantees; third, exploring the application of $A_{3}$ in privacy-preserving machine learning, such as ensuring fairness in federated learning systems where local hardware biases may compromise global model equity.

## Data Availability

The original contributions presented in this study are included in the article. Further inquiries can be directed to the corresponding author.

## Appendix A

**Definition** **A1** (Möbius function)**.** *The* Möbius function μ:N→{−1,0,1} *is defined by*

$$\mu \left(1\right)=1,\;\mu \left(d\right)=\left\{\begin{array}{cc} 0, & \mathrm{if}\;d\;\mathrm{is}\;\mathrm{divisible}\;\mathrm{by}\;p^{2}\;\mathrm{for}\;\mathrm{some}\;\mathrm{prime}\;p,\\ {\left(-1\right)}^{k}, & \mathrm{if}\;d\;\mathrm{is}\;a\;\mathrm{product}\;\mathrm{of}\;k\;\mathrm{distinct}\;\mathrm{primes}. \end{array}\right.$$

*Equivalently,* μ(d)=0 *if d is not square-free, and if d is square-free then* $\mu \left(d\right)={\left(-1\right)}^{\omega (d)}$*, where* ω(d) *denotes the number of distinct prime divisors of d.*
**Lemma A1** (Acceptance probability via Möbius inversion and Fourier bounds)**.** *Let* n≥2*, and let* N∼ Bin (*n,p*) *with* p∈(0,1) *and* q:=1−p*. Define the acceptance probability*

$$\alpha_{n}\;:=\;P\left(\gcd(N,n)=1\right).$$

*Let* μ(·) *be the Möbius function,* φ(·) *Euler’s totient, and* τ(n):=#{d:d∣n} *the divisor-counting function. Then*

$$\alpha_{n}\;=\;\frac{\varphi (n)}{n}\;+\;r_{n},\;\mathrm{with}\;\left|r_{n}\right|\;\leq \;\frac{\sqrt{\pi }}{4\sqrt{pq}}\cdot \frac{\tau (n)}{\sqrt{n}}.$$

*In particular, for all sufficiently large n (depending on p),*

$$\alpha_{n}\;\geq \;\frac{1}{2}\cdot \frac{\varphi (n)}{n}.$$

**Proof.** Step 1: Möbius inversion for the coprimality indicator. For integers k,n≥1 one has the standard identity

$$1\left\{\gcd\left(k,n\right)=1\right\}\;=\;\sum_{d|\gcd(k,n)}\mu \left(d\right)\;=\;\sum_{d|n}\mu \left(d\right)\;1\left\{d|k\right\}.$$

This identity is also valid for k=0. It is important to note the handling of gcd(0,n)=n. Taking expectation with k=N gives

(A1) $$\alpha_{n}\;=\;E\left[1\{\gcd(N,n)=1\}\right]\;=\;\sum_{d|n}\mu \left(d\right)\;P\left(d|N\right).$$

Step 2: Exact Fourier formula for P(d∣N). Fix d≥2. Let $\zeta_{d}:=e^{2\pi i/d}$. By the discrete Fourier inversion on $Z_{d}$,

$$1\left\{d|N\right\}\;=\;\frac{1}{d}\sum_{m=0}^{d-1}\zeta_{d}^{\;mN}.$$

Taking expectation yields

(A2) $$P\left(d|N\right)\;=\;\frac{1}{d}\sum_{m=0}^{d-1}E\left[\zeta_{d}^{\;mN}\right]\;=\;\frac{1}{d}\sum_{m=0}^{d-1}{\left(q+p\;\zeta_{d}^{\;m}\right)}^{n},$$

since $E\left[z^{N}\right]={\left(q+pz\right)}^{n}$ for N∼Bin(n,p). From (A2),

$$P\left(d|N\right)-\frac{1}{d}\;=\;\frac{1}{d}\sum_{m=1}^{d-1}{\left(q+p\;\zeta_{d}^{\;m}\right)}^{n},$$

so by the triangle inequality,

(A3) $$\left|P\left(d|N\right)-\frac{1}{d}\right|\;\leq \;\frac{1}{d}\sum_{m=1}^{d-1}{\left|q+p\;\zeta_{d}^{\;m}\right|}^{n}.$$

Step 3: A uniform bound on ${\left|q+pe^{i\theta }\right|}^{n}$. For θ∈R,

$$|q+pe^{i\theta }{|}^{2}=q^{2}+p^{2}+2pq\cos\theta =1-2pq\left(1-\cos\theta \right).$$

Using $1-x\leq e^{-x}$ and the inequality $1-\cos\theta \geq 2\theta^{2}/\pi^{2}$ for θ∈[0,π], we obtain for θ∈[0,π],

$$|q+pe^{i\theta }{|}^{2}\leq \exp\;\left(-2pq(1-\cos\theta )\right)\leq \exp\;\left(-4pq\;\theta^{2}/\pi^{2}\right),$$

hence

(A4) $$|q+pe^{i\theta }|\leq \exp\;\left(-2pq\;\theta^{2}/\pi^{2}\right),\;\theta \in \left[0,\pi \right].$$

Now in (A3), pair *m* with d−m to reduce to angles in [0,π]:

$$\sum_{m=1}^{d-1}{\left|q+p\zeta_{d}^{\;m}\right|}^{n}=2\sum_{m=1}^{\left\lfloor (d-1)/2\right\rfloor }{\left|q+pe^{2\pi im/d}\right|}^{n},$$

and for 1≤m≤⌊(d−1)/2⌋, $\theta_{m}:=2\pi m/d\in \left(0,\pi \right]$. Applying (A4) yields

$${\left|q+pe^{2\pi im/d}\right|}^{n}\leq \exp\;\left(-2pq\;n\;\theta_{m}^{2}/\pi^{2}\right)=\exp\;\left(-2pq\;n\;{\left(2\pi m/d\right)}^{2}/\pi^{2}\right)=\exp\;\left(-8pq\;n\;m^{2}/d^{2}\right).$$

Therefore,

$$\sum_{m=1}^{d-1}{\left|q+p\zeta_{d}^{\;m}\right|}^{n}\leq 2\sum_{m=1}^{\infty }\exp\;\left(-8pq\;n\;m^{2}/d^{2}\right)\leq 2\int_{0}^{\infty }\exp\;\left(-8pq\;n\;x^{2}/d^{2}\right)\;dx.$$

Evaluating the Gaussian integral gives

$$2\int_{0}^{\infty }e^{-8pq\;n\;x^{2}/d^{2}}\;dx=2\cdot \frac{d}{2}\sqrt{\frac{\pi }{8pq\;n}}=d\cdot \frac{\sqrt{\pi }}{\sqrt{8pq\;n}}.$$

Plugging this into (A3) yields the uniform bound independent of *d*

(A5) $$\left|P\left(d|N\right)-\frac{1}{d}\right|\leq \frac{1}{d}\cdot d\cdot \frac{\sqrt{\pi }}{\sqrt{8pq\;n}}=\frac{\sqrt{\pi }}{\sqrt{8pq}}\cdot \frac{1}{\sqrt{n}}\leq \frac{\sqrt{\pi }}{4\sqrt{pq}}\cdot \frac{1}{\sqrt{n}}.$$

Step 4: Summation over divisors of *n*. Decompose (A1) as

$$\alpha_{n}=\sum_{d|n}\frac{\mu (d)}{d}\;+\;\sum_{d|n}\mu \left(d\right)\left(P\left(d|N\right)-\frac{1}{d}\right).$$

The first sum equals φ(n)/n (a standard identity). For the remainder, use |μ(d)|≤1 and (A5):

$$|r_{n}|\;\leq \;\sum_{d|n}\left|P\left(d|N\right)-\frac{1}{d}\right|\leq \tau \left(n\right)\cdot \frac{\sqrt{\pi }}{4\sqrt{pq}}\cdot \frac{1}{\sqrt{n}}.$$

This proves the stated error bound. Finally, since $\tau \left(n\right)=n^{o(1)}$, the bound implies $|r_{n}|=o\left(1\right)$ as n→∞, and in particular $|r_{n}|\leq \frac{1}{2}\varphi \left(n\right)/n$ for all sufficiently large *n* (depending on *p*), which yields $\alpha_{n}\geq \frac{1}{2}\;\varphi \left(n\right)/n$. □
